# Prognostic Significance of Serum Free Amino Acids in Head and Neck Cancers

**DOI:** 10.3390/cells8050428

**Published:** 2019-05-09

**Authors:** Vit Vsiansky, Marketa Svobodova, Jaromir Gumulec, Natalia Cernei, Dagmar Sterbova, Ondrej Zitka, Rom Kostrica, Pavel Smilek, Jan Plzak, Jan Betka, David Kalfert, Michal Masarik, Martina Raudenska

**Affiliations:** 1 Department of Pathological Physiology, Faculty of Medicine, Masaryk University/Kamenice 5, CZ-625 00 Brno, Czech Republic; vit@vsiansky.cz (V.V.); marketa.sz@seznam.cz (M.S.); j.gumulec@med.muni.cz (J.G.); masarik@med.muni.cz (M.M.); 2 Department of Physiology, Faculty of Medicine, Masaryk University/Kamenice 5, CZ-625 00 Brno, Czech Republic; 3 Department of Chemistry and Biochemistry, Mendel University, Zemedelska 1, 613 00 Brno, Czech Republic; natalia.cernei@centrum.cz (N.C.); dagmar.sterbova@mendelu.cz (D.S.); zitkao@seznam.cz (O.Z.); 4 Central European Institute of Technology, Brno University of Technology, Purkynova 656/123, CZ-612 00 Brno, Czech Republic; 5 Department of Otorhinolaryngology and Head and Neck Surgery, St. Anne’s Faculty Hospital, Pekarska 53, CZ-656 91 Brno, Czech Republic; rom.kostrica@fnusa.cz (R.K.); pavel.smilek@fnusa.cz (P.S.); 6 Department of Otorhinolaryngology and Head and Neck Surgery, University Hospital Motol, First Faculty of Medicine, Charles University, V Uvalu 84, Prague 5, 150 06, Czech Republic; Jan.Plzak@lf1.cuni.cz (J.P.); Jan.Betka@fnmotol.cz (J.B.); David.Kalfert@fnmotol.cz (D.K.); 7 BIOCEV, First Faculty of Medicine, Charles University, Průmyslová 595, 252 50, Vestec, Czech Republic

**Keywords:** head and neck cancer, blood biomarkers, prognosis, colony forming assay, metabolism, amino acids

## Abstract

Despite distinctive advances in the field of head and neck squamous cell cancer (HNSCC) biomarker discovery, the spectrum of clinically useful prognostic serum biomarkers is limited. As metabolic activities in highly proliferative transformed cells are fundamentally different from those in non-transformed cells, specific shifts in concentration of different metabolites may serve as diagnostic or prognostic markers. Blood amino acids have been identified as promising biomarkers in different cancers before, but little is known about this field in HNSCC. Blood amino acid profiles of 140 HNSCC patients were examined using high-performance liquid chromatography. Cox proportional hazards regression model was used to assess the prognostic value of amino acid concentrations in serum. Colony forming assay was used to identify the effect of amino acids that were significant in Cox proportional hazards regression models on colony forming ability of FaDu and Detroit 562 cell lines. In the multivariable Cox regression model for overall survival (OS), palliative treatment was associated with an unfavourable prognosis while high serum levels of methionine have had a positive prognostic impact. In the relapse-free survival (RFS) multivariable model, methionine was similarly identified as a positive prognostic factor, along with tumor localization in the oropharynx. Oral cavity localization and primary radio(chemo)therapy treatment strategy have been linked to poorer RFS. 1mM serine was shown to support the forming of colonies in both tested HNSCC cell lines. Effect of methionine was exactly the opposite.

## 1. Introduction

Metabolomics is a relatively new scientific discipline involving the identification and quantification of the small-molecular metabolites produced by an organism under specific conditions. It seems to be a very promising approach for biomarker research due to the dynamic character of the metabolome that reflects current processes in the body. The specific shifts in concentration of different metabolites could potentially serve as promising diagnostic or prognostic markers of tumorigenesis because metabolic activities in continuously proliferating cells are fundamentally different from those in non-transformed cells and metabolic reprogramming represents a key hallmark of cancer [1]. The specific requirements of tumor cells and up-regulation of some key metabolic enzymes are usually largely reflected in the host organism. Metabolic reprogramming of tumor cells has a severe impact at the systemic level, eventually leading to cancer cachexia [2].

Tumors of the head and neck affect the oral cavity and the upper aerodigestive tract. These tumors most commonly arise from the epithelial layer of mucosal linings. The most frequent histological type of head and neck cancer is squamous cell carcinoma (HNSCC). Nowadays, the prognosis of HNSCC is based on the tumor size and location, the presence of lymph node and distant metastasis. However, there is still a considerable variation in the prognosis within a group of patients sharing the same clinicopathological characteristics [3]. Despite distinctive advances in the field of HNSCC biomarker discovery, no clinically useful prognostic serum biomarkers have been found yet for HNSCC [4]. The tumour growth is dependent on amino acids (AA), which serve as building blocks and a source of cellular energy. Many types of cancer cells overproduce enzymes for amino acid degradation, which boosts anabolic processes but also serves as a mechanism for immune evasion [5] as nutrient-sensing mechanisms orchestrate the action of immune cells in the tumour microenvironment (TME). Tumour-infiltrating immune cells usually suffer from metabolic stress due to abnormal metabolic activity of tumour cells which may result in impaired immune response and immune evasion of tumour cells [6]. Amino acids also have a central role in the maintenance of redox homeostasis in healthy and cancer cells [7]. Patients having the type of tumour which can manipulate amino acid metabolism will probably have changes in AA profiles and also worse prognosis. These patients will probably not benefit from immunotherapy, and competition for AA important for maintaining redox balance can cause more severe damage of healthy cells by chemo- or radiotherapy. Here, we hypothesize that minimally invasive metabolomic approach may provide a relevant method to distinguish clinically important subgroups between HNSCC patients. This study aimed to quantitate free amino acids in human serum and identify potential HNSCC biomarkers. Amino acid profiles were examined by using high-performance liquid chromatography with fluorescence detection. Blood amino acids have been identified as promising biomarkers in different cancers before [8,9,10,11], but little is known about this field in HNSCC.

## 2. Materials and Methods

### 2.1. Patient Selection and Samples Preparation

The study was conducted in accord with the Helsinki Declaration of 1964 and all subsequent revisions thereof. It was approved by the ethical committee of St. Anne’s Faculty Hospital, Brno, and by the ethical committee of University Hospital Motol, Prague, Czech Republic. Blood samples were taken from all patients (Caucasian patients from the Czech Republic diagnosed in the years 2015–2018) indicated for suspicion for HNSCC to biopsy during biopsy procedure after they signed the informed consent. Blood samples were obtained by venipuncture. The blood samples were centrifuged at 3000 rpm at 4 °C for 10 min within 60 min after collection. Serum was aliquoted and stored at −80 °C until analysis. Not all these samples were included into the study.

The inclusion criteria were as follows: histologically verified primary HNSCC, age 40–95 years; no prior anticancer therapy; no diabetes, no uncontrolled hypertension or infections; normal liver, heart and kidney function; and adequate bone marrow reserve.

### 2.2. Amino Acids Profiling

Determination of amino acids was performed by high-performance liquid chromatography with fluorescence detection. An HP 1100 (Hewlett-Packard, Waldbronn, Germany) chromatographic system with a fluorescence detector (FLD) was used. The system was controlled with ChemStation software (rev. A 07.01). The column effluent was monitored with a diode array detector at 338 nm (10 nm bandwidth) and a fluorescence detector at ex/em 340/450 nm and 266/305 nm respectively using the o-phtalaldehyde (OPA) and 9-fluorenylmethyl chloroformate (FMOC) reagents for pre-column derivatization. 

A standard Agilent Technologies procedure (Zorbax Eclipse AAA column, 4.6 × 150 mm, 3.5 mm; mobile phase A, 40 mM Na2HPO4 adjusted to pH 7,8 with 10 M NaOH solution; mobile phase B, ACN/MeOH/water (45:45:10 *v*/*v*/*v*); gradient, from 0 min 0% B, 1.9 min 0% B, 18.1 min 57% B, 18.6 min 100% B, 22.3 min 100% B, 23.2 min 0%B to 26 min; flow rate 2 mL/min; temperature of the column oven, 40 °C) was applied. The concentrations of individual amino acids were calculated based on the calculation of the linear regression equation from constructed calibration curves.

### 2.3. Colony Forming Assay

Colony forming assay is the method of choice to determine cell reproductive capacity after specific treatment (here methionine and serine at 1mM concentration). Only a fraction of seeded cells retains the capacity to produce colonies. HNSCC model FaDu and Detroit 562 cells were harvested with trypsin in EDTA and centrifuged at 2700 rpm/7 min. Cells were then re-suspended in fresh medium and counted using CASY^®^ Cell Counter. Cells were seeded onto a 6-well plate. Each well contained 2 mL media (MEM) and 100, 500, 1000 or 2000 cells. The cells were cultured in the MEM medium with Earle‘s Salts and stable glutamine (Biosera) with 10% FBS and 1% HEPES (Sigma Aldrich, St. Louis, MO, USA) supplemented with antibiotics (penicillin 100 U/mL and streptomycin 0.1 mg/mL; Sigma Aldrich). The cells were maintained at 37 °C in the humidified (60%) incubator with 5% CO2 (Sanyo, Osaka, Japan). Plates were cultivated for 1–3 weeks. Optimal seeding was 500 cells. Any shaking or moving with plates was avoided to obtain clear colonies. Cells were then fixed with cold methanol and visualised with trypan blue. Area of the well covered by colonies was measured on well plate images using a MATLAB script. All colony forming assays were done in triplicates. FaDu is an epithelial adherent cell line derived from squamous cell hypopharyngeal tumour that did not carry PI3K mutations, while Detroit 562 is an epithelial adherent cell line derived from pleural effusion metastasis of pharyngeal carcinoma with the PI3K mutation [12].

### 2.4. Statistical Analysis

Overall survival (OS) was defined as the time in months from the date of initial diagnosis to date of death or the last follow-up visit. Relapse-free survival (RFS) was measured as time in months from the date of initial diagnosis to the date of cancer recurrence, date of death or last follow-up visit. 

Patients were split into groups with high or low various amino acid concentrations in serum based on cut-off points calculated by maximally selected rank statistics. In short, this method establishes a cut-off point from the continuous serum amino acid level data by selecting a value with the highest significance of a standardized log-rank statistic [13]. The minimal possible cut-off value was capped at being higher than at least the lowest 20% of values while the maximal possible value was not larger than the lowest 80% of values.

Univariable, as well as multivariable Cox proportional hazards regression models were used to assess the prognostic value of amino acid concentrations in serum as well as age, sex, tumour localization, T stage (T3,4 vs. T1,2), N stage (N2,3 vs. N0,1), M stage (M1 vs. M0), histological tumour grade and treatment strategy (Palliative; Only Surgery; Surgery with adjuvant radio(chemo)therapy or Primary radio(chemo)therapy). Only those variables which proved to be significant in univariate analysis were selected for the final multivariate model. Those that remained significant in the multivariate analysis were ultimately considered as significant prognostic factors.

Colony forming assay results were analysed using one way ANOVA. Statistical analyses were performed using the R packages survival (version 2.43-1, https://CRAN.R-project.org/package=survival) and maxstat (version 0.7-25, https://CRAN.R-project.org/package=maxstat). Two-sided *p*-values < 0.05 were considered statistically significant.

## 3. Results

### 3.1. Clinicopathological Characterization of HNSCC Patients

In this study, a total of 140 blood samples from patients with histologically verified squamous cell carcinoma and comprehensive patient history were used. Clinicopathological features of the selected patient group are summarized in Table 1.

### 3.2. Association between Amino Acid Serum Levels and Survival

Univariate Kaplan-Meier analysis showed a significant positive correlation of high alanine (HR, 0.4846; 95% CI, 0.2928–0.8019, *p* = 0.00482) and methionine (HR, 0.434; 95% CI, 0.2648–0.7114, *p* ≥ 0.001) serum levels with overall survival (OS). On the other hand, high levels of cystine (HR, 1.684; 95% CI, 1.043–2.721, *p* = 0.0331) predicted lower OS. Several clinical parameters, namely tumor location in hypopharynx (HR, 2.985; 95% CI, 1.72–5.18, *p* > 0.001), higher T stage (T 3,4 vs. T 1,2; HR, 1.633; 95% CI, 1.003–2.659, *p* = 0.0486), higher N stage (N 2,3 vs. N 0,1; HR, 1.898; 95% CI, 1.184–3.041, *p* = 0.00776), primary radio(chemo)therapy treatment modality (HR, 1.703; 95% CI, 1.067–2.72, *p* = 0.0258) and palliative treatment (HR, 6.483; 95% CI, 3.552–11.83, *p* ≥ 0.001) also proved to be significant negative predictors of OS. Oropharyngeal tumor location (HR, 0.566; 95% CI, 0.337–0.9505, *p* = 0.0314) and surgical treatment with adjuvant radio(chemo)therapy (HR, 0.2778; 95% CI, 0.1457–0.5296, *p* ≥ 0.001) were associated with positive impact on OS.

Further univariate analysis of relapse-free survival (RFS) revealed three significant protective factors: oropharyngeal tumor location (HR, 0.1996; 95% CI, 0.06907–0.5768, *p* = 0.00292), high serum levels of threonine (HR, 0.209; 95% CI, 0.04956–0.8813, *p* = 0.033), and methionine (HR, 0.3756; 95% CI, 0.1726–0.8175, *p* = 0.0136). Conversely, tumor location in oral cavity (HR, 2.72; 95% CI, 1.153–6.415, *p* = 0.0223) and primary radio(chemo)therapy (HR, 2.503; 95% CI, 1.17–5.359, *p* = 0.0181) predicted worse RFS.

### 3.3. Multivariate Analysis

**Figure 1:**
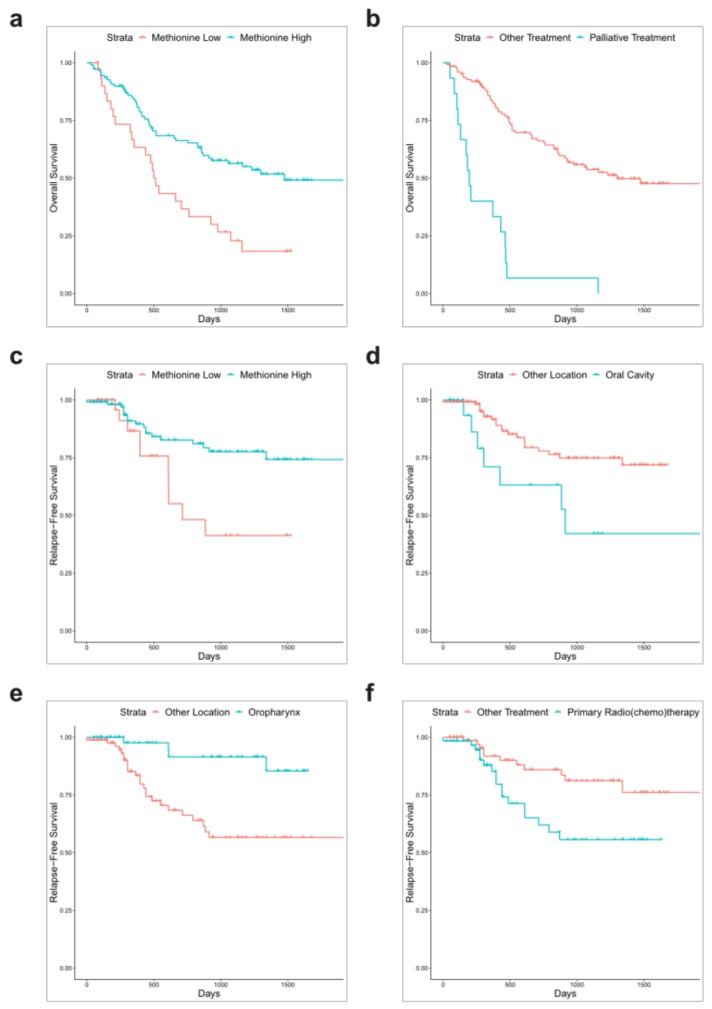
Kaplan-Meier plots for 5-year overall survival (OS) and relapse-free survival (RFS) stratified by statistically significant factors in the multivariate regression analysis: Serum concentration of methionine and overall survival (OS) (**a**), palliative treatment and OS (**b**), serum concentration of methionine and relapse-free survival (RFS) (**c**), oral cavity location and RFS (**d**), oropharyngeal location and RFS (**e**) and primary radio(chemo)therapy treatment and RFS (**f**). Data for statistically insignificant factors in the multivariable model are not shown.

Statistically significant variables from the univariate analyses were used to create a multivariate Cox regression model both for OS and RFS. In this model for OS, palliative treatment (HR, 3.6380; 95% CI, 1.2499–10.5885, *p* = 0.0178) remained a significant negative prognostic factor. A single amino acid was found to be a positive prognostic factor for OS in this model, namely methionine (HR, 0.5248; 95% CI, 0.3056–0.9013, *p* = 0.0195); see Figure 1. Goodness-of-fit of this model was evaluated by several statistical methods: concordance (0.757; Standard error = 0.027), likelihood ratio test (62.4 with 10 degrees of freedom; *p* > 0.001) and Wald test (61.79 with 10 degrees of freedom; *p* > 0.001). Two positive prognostic factors remained statistically significant in the RFS multivariate regression analysis: oropharyngeal tumor location (HR, 0.2998; 95% CI, 0.09951–0.9034, *p* = 0.03232) and high levels of methionine (HR, 0.4373; 95% CI, 0.19240–0.9941, *p* = 0.04836). Oral cavity tumor location (HR, 4.6898; 95% CI, 1.49124–14.7489, *p* = 0.00820) and primary radio(chemo)therapy (HR, 5.1464; 95% CI, 1.89193–13.9992, *p* = 0.00133) both predicted worse RFS, same as in univariable analysis; see Figure 1. Concordance of this model was 0.787 (Standard error = 0.043), likelihood ratio test statistic equaled 33.05 (*p* > 0.001) and Wald test statistic was 24.63 (*p* > 0.001). All statistically significant results of the univariate survival analysis along with multivariate analysis are briefly summarized in Table 2. Complete data and results of the Cox regression model are available in Tables S1–S6.

### 3.4. Colony-Forming Capacity

**Figure 2:**
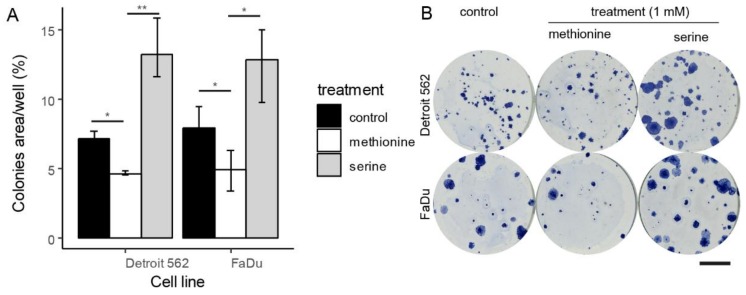
Colony-forming assays, in vitro cell line experiment on head and neck squamous cell cancer (HNSCC) primary (FaDu) and secondary (Detroit 562) cell lines. Shown area percentage of well area covered by cells after treatment in concentration 500 cells/well. (**A**) displayed as median and interquartile range, * indicate *p* < 0.05, ** *p* < 0.001 (**B**) representative wells, calibration chart denotes 10 mm.

In the multivariate Cox regression model for OS and RFS, high methionine serum levels turned out to be a significant positive prognostic factor. On the other hand, no studied amino acid was found to be a significant negative prognostic factor for OS or RFS in this model. Nevertheless, serine serum levels showed borderline significance for OS in univariate analysis and serine has a widely accepted role in cancer cell metabolism. Therefore, colony-forming assays with non-toxic (based on MTT) 1mM concentrations of serine and methionine were performed to measure the proliferative capacity of cancer cells after treatment through their ability to form colonies. This assay can distinguish cells that are viable and able to divide. As a model of HNSCC, primary tumour-derived FaDu and metastasis-derived Detroit 562 cell lines were used. 1 mM serine was shown to support the forming of colonies in both tested HNSCC cell lines (4% and 6% growth increase at *p* = 0.05 and < 0.001 for FaDu and Detroit 562, respectively). Effect of methionine was exactly the opposite (4% and 3% growth decrease at *p* = 0.05 and 0.04 for FaDu and Detroit). In conclusion, serine supported the growth and division of model cancer cells and methionine reduced the number of proliferative cancer cells. Results of colony-forming assays are summarized in Figure 2.

## 4. Discussion

Cancer cells are champions in acquiring necessary nutrients from a nutrient-poor environment. The metabolic reprogramming associated with cancer has a deep impact on gene expression, cellular differentiation, and the permissiveness of tumour microenvironment [14]. There are many possible mechanisms that may affect free serum amino acid levels in HNSCC patients. First, HNSCC patients are often malnourished because of alcohol abuse and poor dietary habits [15]. Second, cancer cells often exhibit dysregulated expression of amino acid transporters. For example, L-neutral amino acid transporter 1 (LAT1) and system ASC amino-acid transporter-2 (ASCT2) are highly expressed in tongue cancer and can serve as prognostic factors for predicting worse outcome after surgical treatment [16]. A third possible mechanism is a change in the immune system and intestinal function due to cancer development and/or treatment [17]. For example, most of the circulating citrulline is derived from glutamine conversion in enterocytes [18] and low blood citrulline level may point to mucosal barrier injury caused by chemotherapy or radiotherapy [19,20]. Accordingly, specific shifts in the concentration of different metabolites could occur due to cancer progression.

This study has evaluated the amino acid profiles in the serum of HNSCC patients as possible prognostic markers in HNSCC. Young HNSCC patients were excluded from this study because HNSCC in young patients often occurs without significant exposure to alcohol and tobacco but rather due to severe genetic predisposition. Metabolic changes may not play a major role in these cases [21]. In multivariate analysis (Cox) for OS, palliative treatment was found to be a significant negative prognostic factor and methionine was found to be a positive prognostic factor. Two positive prognostic factors remained statistically significant in the RFS multivariate regression analysis: oropharyngeal tumor location and high serum levels of methionine. Oral cavity tumour location and primary radio(chemo)therapy both predicted worse RFS, same as in univariable analysis. The unfavourable prognosis of oral cavity tumours was also described in [22]. 

A single amino acid was found to be a positive prognostic factor for both OS and RFS in our model, namely methionine. Many studies have reported that methionine plays a key role in antioxidant processes and has a protective effect against cancer. Chronic deprivation of methionine has been associated with tumour development [23,24,25]. Low levels of methionine also lead to DNA hypomethylation which is typical in malignant cells [26], since methionine is the primary source of methyl groups [27]. Nevertheless, methionine tumour promoting effect and methionine dependency has been observed in many types of cancer [27,28]. For example, PI3K oncogenic mutations have been associated with development of methionine dependency [29]. Analysis of whole-exome sequencing data revealed that nearly one-third of HNSCC tumours have PI3K pathway mutations and all tumours with concurrent mutation of multiple PI3K pathway genes were advanced [30]. Accordingly, high serum levels of methionine can implicate the absence of such kind of driver mutation and indicate a better prognosis. Furthermore, methionine levels were found to be lower in patients with a relapse of oral cancer than in those without relapse [31]. 1mM methionine was shown to dampen the formation of colonies in both tested HNSCC cell lines in our study. High levels of threonine and high levels of alanine were shown to be beneficial for RFS and OS, respectively (univariate Cox analysis). Higher threonine levels may be associated with good intestinal function because dietary threonine is preferentially used for mucin synthesis in gastrointestinal tissues [32,33,34]. Dysbiosis and loss of protective mucus are important effects of alcohol abuse and poor dietary habits and may be further aggravated by chemotherapy and radiotherapy [35,36]. There are many mechanisms by which gut microbiota dysbiosis causes elevated intestinal permeability, aberrant immune activation, and chronic inflammation. All of them may contribute to cancer relapse and progression [37]. The decrease of threonine was also observed in patients with pancreatic cancer [38].

No studied amino acid was found to be a significant negative prognostic factor for OS or RFS in multivariate analysis. Nevertheless, serine serum levels showed borderline significance for OS in the univariate analysis and serine has a widely accepted role in cancer cell metabolism. Changes in serine metabolism have long been observed in malignant cells because serine serves as a central hub in the metabolic network for many aspects of cancer cell survival and proliferation [14,39,40]. Higher concentrations of serine were detected in the malignant HNSCC tissue samples compared to surrounding normal tissues [31,41]. Recently, Gao et al. found that serine-derived lipids and ceramides are essential for mitochondrial function because serine deficiency caused mitochondrial fragmentation [42]. High accessibility of blood serine may, therefore, be important for circulating cancer cells because they exhibit enhanced mitochondria biogenesis and OXPHOS-dependent metabolism [43]. Furthermore, mitochondrial metabolism is required for maintenance of cancer stem cells [44]. Accordingly, 1mM serine was shown to support the forming of colonies in both tested HNSCC cell lines in our study. High circulating serine levels were also observed in pancreatic, lung and breast cancers [38,45,46,47]. Some studies show significant reduction of preoperative serum levels of almost all amino acids except cystine in HNSCC patients compared with the healthy control group [15], but this is rather the result of severe malnutrition of HNSCC patients. 

As HNSCC tumors are both biologically and clinically heterogeneous and our regression models lack external validation, it must be noted that we view our results as a prospective clinical study backed by in vitro experiments that can hopefully lay a foundation for larger investigations in the future.

## Figures and Tables

**Table 1:** Clinicopathological features of the selected patient group (TNM 7th edition was used).

Parameter	Level of Parameter	n
Sex		
	Male	133
	Female	7
Median Age (Range)		67 (48–93)
Tumor Location		
	Oral Cavity	20
	Oropharynx	54
	Hypopharynx	23
	Larynx	43
Differentiation		
	Low Grade	8
	Intermediate Grade	101
	High Grade	31
T stage		
	T1	17
	T2	44
	T3	29
	T4	50
N stage		
	N0	52
	N1	19
	N2	60
	N3	9
M stage		
	M0	135
	M1	5
Treatment		
	Surgery	18
	Surgery with adjuvant RT/CT	44
	Primary RT/CT	63
	Palliative	15

**Table 2:** Survival analysis.

Parameter	Univariate	Multivariate	
	*p*	HR (95% CI)	*p*
**OS**			
Hypopharynx	>0.001	1.3596 (0.7103–2.6025)	0.3538
Oropharynx	0.0314	0.7232 (0.3910–1.3376)	0.3017
T 3,4 vs. T 1,2	0.0486	1.5954 (0.9483–2.6843)	0.0784
N 2,3 vs. N 0,1	0.00776	1.7314 (0.9869–3.0374)	0.0556
Palliative Treatment	>0.001	3.6380 (1.2499–10.5885)	0.0178
Primary RT/CT	0.0258	1.1562 (0.4655–2.8715)	0.7545
Surgery with adjuvant RT/CT	>0.001	0.3771 (0.1375–1.0338)	0.0580
Alanine	0.00482	0.7182 (0.4138–1.2467)	0.2395
Cystine	0.0331	1.0765 (0.6204–1.8679)	0.7932
Methionine	>0.001	0.5248 (0.3056–0.9013)	0.0195
RFS			
Oral Cavity	0.0223	4.6898 (1.49124–14.7489)	0.00820
Oropharynx	0.00292	0.2998 (0.09951–0.9034)	0.03232
Primary RT/CT	0.0181	5.1464 (1.89193–13.9992)	0.00133
Threonine	0.033	0.2893 (0.06630–1.2623)	0.09892
Methionine	0.0136	0.4373 (0.19240–0.9941)	0.04836

OS, 5-year overall survival (OS); RFS, 5-year relapse free survival; HR, hazard ratio.

## Supplementary Materials

The following are available online at https://www.mdpi.com/2073-4409/8/5/428/s1, Table S1: Results of the univariate Cox regression OS analysis for clinicopathological parameters. Table S2: Results of the univariate Cox regression OS analysis for serum amino acid levels. Table S3: Results of the multivariate Cox regression OS analysis for factors identified as significant in the univariate analyses. Table S4: Results of the univariate Cox regression RFS analysis for clinicopathological parameters. Table S5: Results of the univariate Cox regression RFS analysis for serum amino acid levels. Table S6: Results of the multivariate Cox regression RFS analysis for factors identified as significant in the univariate analyses. Dataset containing analysis of free serum amino acid concentrations in patients with head and neck squamous cell carcinoma used in our study is available for download in the Zenodo repository (https://zenodo.org/, Digital Object Identifiers: http://doi.org/10.5281/zenodo.2547469).
