# Supplementary material for: Prognostic Significance of Serum Free Amino Acids in Head and Neck Cancers
Source: Cells. 2019 May 9;8(5):428. doi: 10.3390/cells8050428 (PMC6562773; doi:10.3390/cells8050428)
Supplement: Supplementary file 1 [file cells-08-00428-s001.pdf]

Supplementary information for:

Prognostic significance of serum free amino acids in head and neck cancers

Vit Vsiansky[1×], Marketa Svobodova[1,2×], Jaromir Gumulec[1,2,3], Natalia Cernei[3,4], Dagmar Sterbova[3], Ondrej Zitka[3,4], Rom Kostrica[5], Jan Plzak[6], Jan Betka[6], Michal Masarik[1,2,7], and Martina Raudenska[2*]

[1] Department of Pathological Physiology, Faculty of Medicine, Masaryk University / Kamenice 5, CZ-625 00 Brno, Czech Republic
[2] Department of Physiology, Faculty of Medicine, Masaryk University / Kamenice 5, CZ-625 00 Brno, Czech Republic
[3] Department of Chemistry and Biochemistry, Mendel University, Zemedelska 1, 613 00 Brno, CZ
[4] Central European Institute of Technology, Brno University of Technology, Technicka 3058/10, CZ-616 00 Brno, Czech Republic
[5] Department of Otorhinolaryngology and Head and Neck Surgery, St. Anne's Faculty Hospital, Pekarska 53, CZ-656 91 Brno, Czech Republic
[6] Department of Otorhinolaryngology and Head and Neck Surgery, University Hospital Motol, First Faculty of Medicine, Charles University, V Uvalu 84, Prague 5, 150 06, Czech Republic
[7] BIOCEV, First Faculty of Medicine, Charles University, Průmyslová 595, 252 50, Vestec, Czech Republic
* Correspondence: m.raudenska@gmail.com; Tel.: +420-5-4949-8526

Table S1: Results of the univariate Cox regression overall survival analysis for clinicopathological parameters. Statistically significant values are written in bold.

	Confidence interval	P
Age (>Median)	0.9823 - 1.036	0.525
Sex (Male)	0.4438 - 4.495	0.559
Grade (High)	0.3252 - 2.352	0.791
Grade (Intermediate)	0.2279 - 1.451	0.241
Larynx	0.4249 - 1.198	0.201
Oral Cavity	0.8168 - 2.724	0.193
**Hypopharynx**	**1.72 - 5.18**	**>0.001**
**Oropharynx**	**0.337 - 0.9505**	**0.0314**
**T 3,4 vs. T 1,2**	**1.003 - 2.659**	**0.0486**
**N 2,3 vs. N 0,1**	**1.184 - 3.041**	**0.00776**
M1 vs. M0	0.4144 - 4.197	0.639
**Treatment: Palliative**	**3.552 - 11.83**	**>0.001**
**Treatment: Primary Radio(chemo)therapy**	**1.067 - 2.72**	**0.0258**
Treatment: Surgery	0.2681 - 1.279	0.179
**Treatment: Surgery With Adjuvant Radio(chemo)therapy**	**0.1457 - 0.5296**	**>0.001**

Table S2: Results of the univariate Cox regression overall survival analysis for serum amino acid levels. Statistically significant values are written in bold.

	Confidence interval	P
Aspartic Acid	0.5015 - 1.462	0.57
Glutamic Acid	0.4724 - 1.199	0.232
Asparagine	0.8609 - 2.187	0.183
Serine	0.938 - 2.603	0.0865
Glutamine	0.7799 - 2.11	0.327
Histidine	0.3877 - 1.019	0.0598
Glycine	0.7542 - 2	0.409
Threonine	0.3814 - 1.272	0.239
Citrulline	0.3892 - 1.054	0.0795
Arginine	0.7524 - 2.244	0.348
**Alanine**	**0.2928 - 0.8019**	**0.00482**
Tyrosine	0.5309 - 1.368	0.508
**Cystine**	**1.043 - 2.721**	**0.0331**
Valine	0.4348 - 1.108	0.126
**Methionine**	**0.2648 - 0.7114**	**>0.001**
Tryptophan	0.7276 - 2.047	0.45
Phenylalanine	0.779 - 2.135	0.323
Isoleucine	0.2994 - 1	0.0501
Leucine	0.4636 - 1.241	0.272
Lysine	0.7339 - 2.008	0.45
Proline	0.433 - 1.108	0.126

Table S3: Results of the multivariate Cox regression overall survival analysis for factors identified as significant in the univariate analyses. Statistically significant values are written in bold. Concordance index of this model was 0.757 (Standard error = 0.027); Likelihood ratio test = 62.4 (p > 0.001); Wald test = 61.79 (p > 0.001).

	Confidence interval	P
T 3,4 vs. T 1,2	0.9483 - 2.6843	0.0784
N 2,3 vs. N 0,1	0.9869 - 3.0374	0.0556
Hypopharynx	0.7103 - 2.6025	0.3538
Oropharynx	0.3910 - 1.3376	0.3017
**Treatment: Palliative**	**1.2499 - 10.5885**	**0.0178**
Treatment: Primary Radio(chemo)therapy	0.4655 - 2.8715	0.7545
Treatment: Surgery With Adjuvant Radio(chemo)therapy	0.1375 - 1.0338	0.0580
Alanine	0.4138 - 1.2467	0.2395
Cystine	0.6204 - 1.8679	0.7932
**Methionine**	**0.3056 - 0.9013**	**0.0195**

Table S4: Results of the univariate Cox regression relapse-free survival analysis for clinicopathological parameters.

	Confidence interval	P
Age (>Median)	0.9807 - 1.068	0.287
Sex (Male)	0.2188 - 11.9	0.639
Grade (High)	0.08681 - 1.538	0.170
Grade (Intermediate)	0.11508 - 1.323	0.131
Larynx	0.6918 - 3.094	0.319
**Oral Cavity**	**1.153 - 6.415**	**0.0223**
Hypopharynx	0.7505 - 5.472	0.163
**Oropharynx**	**0.06907 - 0.5768**	**0.00292**
T 3,4 vs. T 1,2	0.341 - 1.509	0.381
N 2,3 vs. N 0,1	0.3982 - 1.881	0.715
M1 vs. M0	0.1677 - 9.101	0.836
Treatment: Palliative	0.128 - 7.149	0.965
**Treatment: Primary Radio(chemo)therapy**	**1.17 - 5.359**	**0.0181**
Treatment: Surgery	0.1047 - 1.864	0.266
Treatment: Surgery With Adjuvant Radio(chemo)therapy	0.2305 - 1.194	0.124

Table S5: Results of the univariate Cox regression relapse-free survival analysis for serum amino acid levels. Statistically significant values are written in bold.

	Confidence interval	P
Aspartic Acid	0.2498 - 1.222	0.143
Glutamic Acid	0.3578 - 1.583	0.454
Asparagine	0.4556 - 2.079	0.944
Serine	0.5217 - 2.902	0.636
Glutamine	0.3749 - 1.678	0.544
Histidine	0.4971 - 3.028	0.657
Glycine	0.3953 - 2.039	0.797
**Threonine**	**0.04956 - 0.8813**	**0.033**
Citrulline	0.238 - 1.17	0.116
Arginine	0.2416 - 2.014	0.506
Alanine	0.2174 - 1.065	0.0711
Tyrosine	0.3283 - 1.459	0.334
Cystine	0.615 - 3.016	0.446
Valine	0.448 - 2.01	0.891
**Methionine**	**0.1726 - 0.8175**	**0.0136**
Tryptophan	0.6011 - 3.101	0.457
Phenylalanine	0.6757 - 3.314	0.32
Isoleucine	0.269 - 1.645	0.377
Leucine	0.4292 - 2.216	0.952
Lysine	0.5153 - 2.523	0.746
Proline	0.2581 - 1.162	0.117

Table S6: Results of the multivariate Cox regression relapse-free survival analysis for factors identified as significant in the univariate analyses. Concordance index of this model was 0.787 (Standard error = 0.043); Likelihood ratio test = 33.05 (p > 0.001); Wald test = 24.63 (p > 0.001).

	Confidence interval	P
**Oral Cavity**	**1.49124 - 14.7489**	**0.00820**
**Oropharynx**	**0.09951 - 0.9034**	**0.03232**
**Treatment: Primary Radio(chemo)therapy**	**1.89193 - 13.9992**	**0.00133**
Threonine	0.06630 - 1.2623	0.09892
**Methionine**	**0.19240 - 0.9941**	**0.04836**
